# Universal confined tensile strength of intact rock

**DOI:** 10.1038/s41598-019-42698-6

**Published:** 2019-04-16

**Authors:** Hengxing Lan, Junhui Chen, Renato Macciotta

**Affiliations:** 1 State Key Laboratory of Resources and Environmental Information System,Institute of Geographic Sciences and Natural Resources Research, Chinese Academy of Sciences, 11A, Datun Road, Chaoyang District, Beijing, 100101 China; 2 School of Geological Engineering and Geomatics, Chang’an University, Xi’an, 710054 China; 3 University of Chinese Academy of Sciences, Datun Road 3A, Beijing, 100049 China; 4 School of Engineering Safety and Risk Management, Faculty of Engineering, University of Alberta, 13-244, Donadeo ICE Building, 9211 – 116 St., Edmonton, AB T6G 1H9 Canada

## Abstract

Strength criteria for intact rock are essential for the safe design of many engineering structures. These criteria have been derived mainly from tests in the compressive stress region. Very few results have been published for confined, direct tensile tests on intact rock. No appropriate criteria are available for addressing the issue on tensile strength of intact rock at current stage. We present the results of direct triaxial tensile tests on Longmaxi Shales under varying confining stresses. These and the results from previous tests in marble and sandstone prove that the phenomenon of “tension cut-off” at low confining stress and the positive correlations between confining stress and tensile strength above the confining stress threshold for brittle rocks occur also in more ductile rocks like shales. Such findings are consistent with the concept that tensile failure processes for intact rock are universal. Our results demonstrate that friction processes still have a significant role on intact rock strength in the tensile region which is leading to confined tensile failure and transitioning to a purely tensile mode. Further, strength criteria are presented which consider the frictional processes leading to failure under confined, direct tension tests and validated against published tensile strength data.

## Introduction

Design and assessment of engineering structures in rock materials requires adopting a set of rock strength criteria^1,2^. Underperformance and collapse are associated with stresses reaching or exceeding these strength criteria^3–5^ and the issue becomes the adequacy of the selected criterion, which can over or under predict rock strength when applied under conditions that are different than those used to derive the criteria^6^. The most common strength criteria used for rock are the Mohr-Coulomb and Hoek-Brown^4^. The Mohr-Coulomb criteria has been used extensively for different materials, including soil, metal and rock^7–10^. The Hoek-Brown criteria has become the preferred criteria for researchers and practitioners to better capture the strength characteristics of rock under varying stresses^11^. However, these criteria have been developed for compressive stress regions following loading stress paths (in the laboratory) as opposed to most engineering projects, which follow unloading stress paths tending towards the tensile region^11^. As a consequence, these criteria do not provide adequate characterization of intact rock strengths in the tensile region^12^ as they do not capture the processes of rock fracturing in tension. In this regard, Hoek and Martin^13^ proposed the use of a tension cut-off based on the Hoek-Brown criterion, which requires specimen testing within the tensile region and transitioning towards the unconfined compressive strength. The authors believe there is an opportunity for improving this approach and the efficiency for estimating the tension cut-off.

Previous research has been done in intact rock samples through indirect tensile laboratory tests^14–16^. These require conversion into applied tension, which is associated with limitations in their interpretation^17–19^. Very few research has been conducted through direct tensile testing, particularly under varying confining stresses. The work of Ramsey and Chester^20^ and Bobich^21^ present direct tensile results on marble and sandstone under different confinement stresses. Their results show the transition of intact rock strength, from a state where they are highly influenced by frictional processes (positive correlation between confining and tensile applied stresses), towards a state where frictional processes do not appear to influence the strength of the materials but are dominated by rock grain strengths and the bond between grains. The question remains about the universality of frictional processes in the tensile region for other rock types that are known to show different behavior from brittle rocks (e.g. shales). We have conducted a new set of direct tensile tests under varying confining stresses on a different rock type, Longmaxi Shale. Tests have expanded the confining stresses applied to the shale samples to include unconfined direct tension. Our results, together with the results for the other rock types, allow for increased understanding of the processes leading to intact rock failure in the tensile region. Moreover, evidence of the transition between frictional processes and pure tension in the tensile region has been acquired through Nano-scale micro-fracture inspection using high-resolution Scanning Electron Microscope (SEM) imaging. Our results also present the opportunity to derive strength criteria considering frictional processes and the transition to a purely tensile mode of failure.

## Confined tensile tests on Longmaxi Shale and previous tests

### Experimental approach

**Figure 1:**
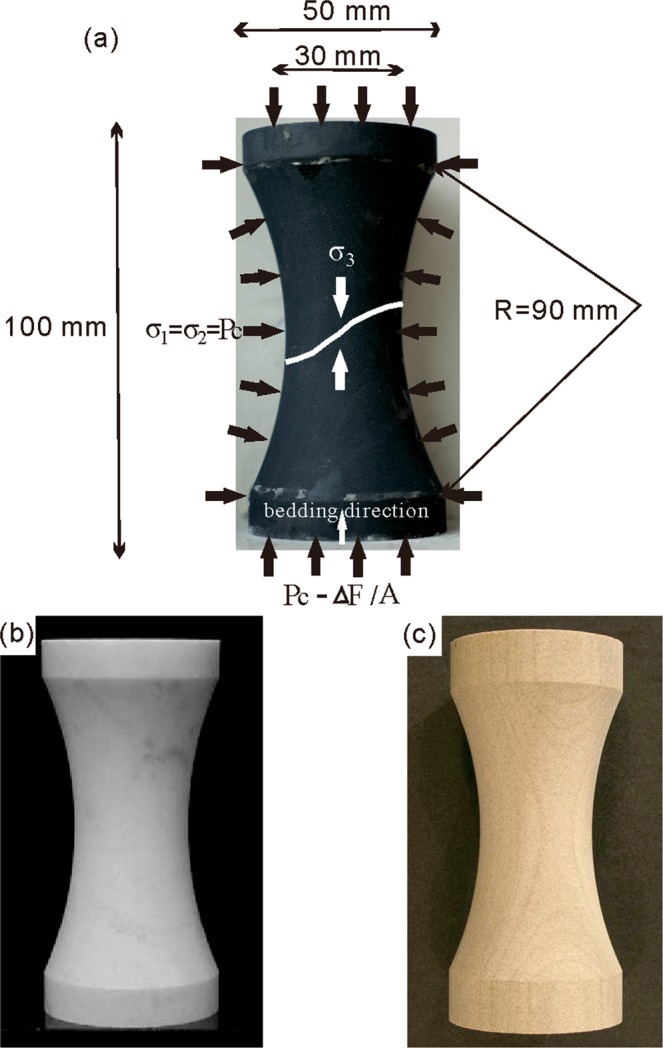
Sample geometry and dimensions. The applied stresses during testing are also shown in (**a**): Pc is the confining pressure, σ_1_, σ_2_ and σ_3_ are the principal stresses (σ_1_ the largest and equal to σ_2_, σ_3_ the lowest) applied at the centre of the specimen, ΔF is the incremental change in tensile force, and A is the section area at the ends of the sample. Bedding direction is shown (vertical direction) and a typical fracture profile after testing is sketched in white over the sample photo. The bottom is sample of (**b**) marble published in Ramsey and Chester^20^ and (**c**) sandstone in Bobich^21^.

We followed the procedures outlined in Ramsey^22^ and Bobich^21^. The samples were obtained as 50 mm diameter cylinders, then grinded to a reduced central neck (known as a dog-bone sample) of 30 mm in diameter following a radius of 90 mm (Fig. 1). The final sample heights were 100 mm. This geometry allows reproducible and uniform stress conditions, and induces the location of failure at the center of the neck (smallest diameter)^22,23^. The rock tested was Longmaxi Shale, characterized by consistent discontinuities (bedding planes) and a strength under unconfined compression of 73.3 MPa. The sample was obtained with its symmetry axis parallel to the orientation of the bedding planes. It is noted that Patel and Martin^24^ showed through axisymmetrical elastic analyses that the vertical tensile stresses within this geometry and at the center of the neck could vary +/− 60% of the average value, with the largest tensile stresses at the surface of the specimen and the lower tensile stresses at the center of the specimen. Calculations here considered the average values at the neck of the specimen and therefore the results present the average conditions at the specimen scale. This is compatible with interpretation for Brazilian Tests and flattened Brazilian Tests for tensile strength, as also shown by Patel and Martin^24^.

Tension loading was applied with a closed-loop computerized triaxial testing apparatus with an integral rigidity of 11 × 10^9^ N/m and servo-hydraulic actuators (MTS 815). The maximum load capacity is 2600 kN with a measuring accuracy of ±0.5%. The sample is placed inside a thick-walled pressure vessel and confinement is applied by injecting pressurized fluid into the vessel. The apparatus required some modifications for the confined tensile tests: (1) Placement of a synchronous chain between the actuator and loading platform. The plates that come in contact with the sample were then fixed to the actuator and platform, respectively, such that displacements can be detected when expanding the system; (2) A sample fixture with multi-degree of freedom was developed that consisted of upper and lower cushion blocks connected to the frame through pistons. The system provides force adjustments if bending is detected. Testing was conducted at room temperature (28 °C) and humidity. Tensile loading was applied through axial displacements at a rate of 2 × 10^−2^ mm/s.

Scanning Electron Microscope (SEM) imaging was used to observe the micro-fracturing of the samples tested. SEM imaging was done using a Zeiss Merlin SEM equipped with secondary electron detectors for Nano-scale micro-fracture imaging (10 nm pixel size).

### Longmaxi Shale test Results and previous tests

Confined tests were conducted on 11 samples of Longmaxi Shale. These covered the tensile stress region (σ_1_ zero and above, σ_3_ below zero) and were extended into the compressive region (σ_1_ above zero, σ_3_ zero and above). σ_1_ was given by the confining stress and σ_3_ was calculated as the confined stress minus the applied axial stresses during the test (ΔF/area of the sample neck). The peak tensile strength obtained was −13.5 MPa at a confining stress of 5 MPa. The tensile strength under no confinement was −5.5 MPa. The maximum strength obtained in the compressive stress region was 50 MPa (σ_1_) at σ_3_ of 22 MPa. Tensile strength data for Carrara Marble and Berea Sandstone were obtained from the literature^20,21^ to complement the tensile strength results obtained for Longmaxi Shale.

**Figure 2:**
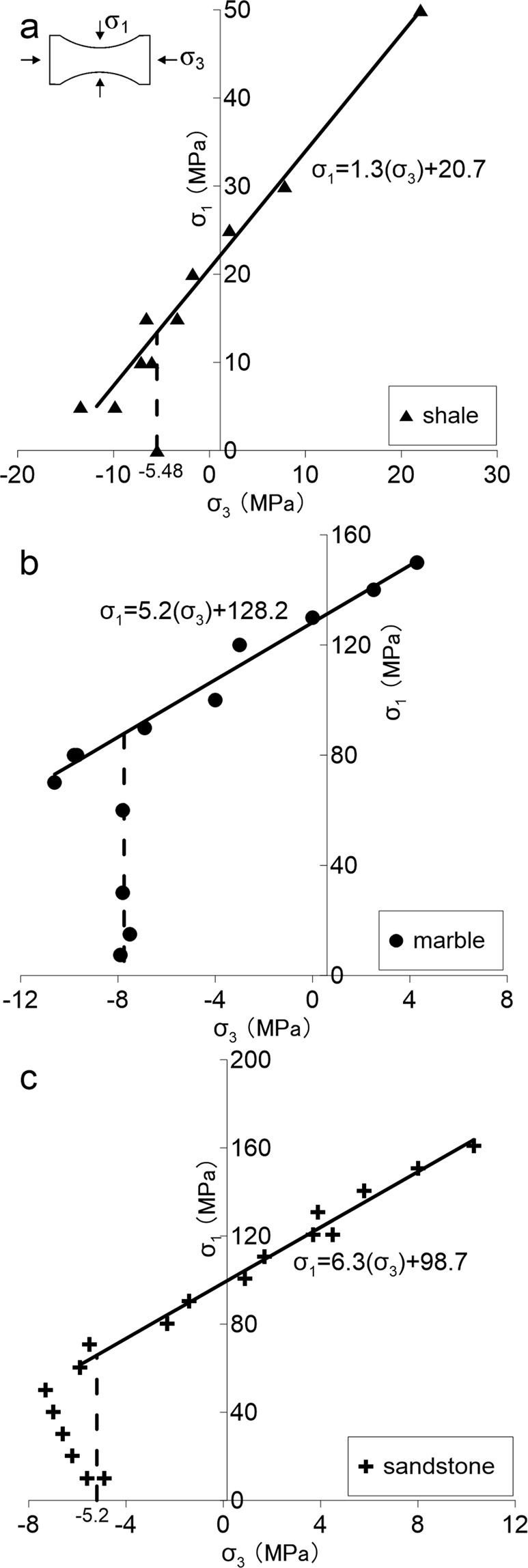
Test results in (**a**) Longmaxi Shale, (**b**) Carrara Marble^20^ and (**c**) Berea Sandstone^21^. Positive trends between confinement and strength are evident for a minimum confinement stress and above, consistent with previous criteria. Tensile strength reduction below this minimum confinement suggests a stress regime where friction has less influence on strength. A tensile cut-off (as shown) can be adopted for strength criteria based on these results.

Figure 2 shows the testing results for Longmaxi Shale as well as the results for Carrara Marble and Berea Sandstone in σ_1_ against σ_3_ plots. This figure shows linear and positive correlations between the applied σ_1_ and σ_3_ for the three rock types. This correlation holds true for the tests with σ_1_ above a threshold value (approximately 5 MPa, 70 MPa and 50 MPa for the shale, marble and sandstone, respectively). These positive correlations are consistent with the concept that strength is a function of confining stress, and would suggest that friction processes have a major effect on the tensile strength of these materials above these thresholds of σ_1_. For lower values of σ_1_, the linear correlation breaks and the tensile strength drops towards the unconfined tensile strength (σ_1_ = 0), suggesting that friction processes are less important and failure is dominated by the strength of the particles and their bonds. This behavior is known as the “tension cut”^13,25^, alluding to the sudden deviation from the positive correlation between σ_1_ and σ_3_, toward the unconfined tensile strength. It is interesting to note that the “tension cut-off” remains true for Logmaxi Shale, indicating this cut is a universal behavior for intact rock.

### Fracturing and strength in the tensile region

**Figure 3:**
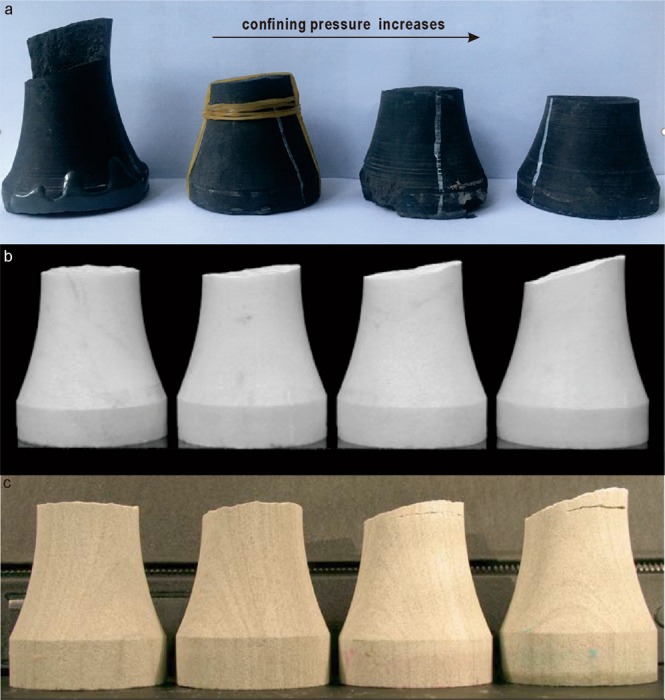
Fracture angle of Longmaxi Shale (**a**), Carrara Marble^20^ (**b**), and Berea Sandstone^21^ (**c**). From left to right, the fracture angles for marble and sandstone increase with confining stress. The fracture angle for the shale, on the contrast, decreases as confining stress increases.

**Figure 4:**
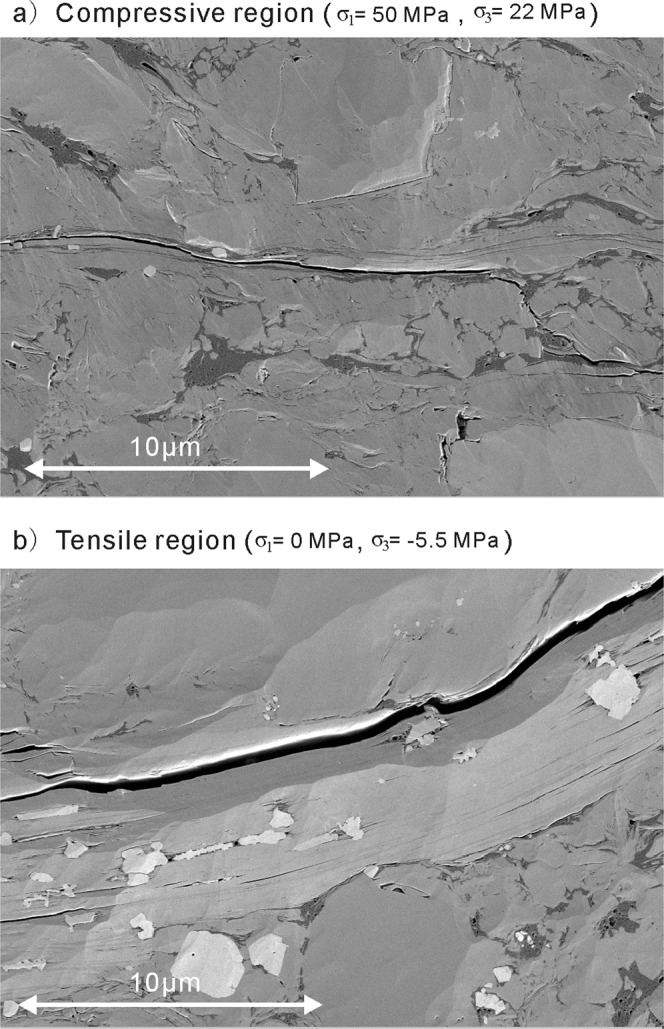
Scanning Electron Microscope (SEM) imaging of typical micro-fractures in Longmaxi Shale after testing in the compressive region (**a**) and the tensile region (**b**). Images evidence the lack of contact (larger fracture aperture) between fracture surfaces in the sample tested under the lowest confining stresses, limiting the ability for mobilizing friction processes.

Martin^26^ and Rafiei Renani and Martin^27^ showed that failure of intact rock is initiated by the formation of new fractures (crack initiation), followed by progressive growth and further coalescence of the fractures until a critical condition is reached (crack damage) and total collapse. Therefore, the effect of friction by the time of failure will depend on the availability of these fractures to shear, and the stresses acting on their surfaces. Higher confining stresses would promote fracture surface contact and increased shear strength, while no confinement would be associated with lack of contact at the fracture surfaces or low stresses at these contacts. Figure 3 shows the fracture angle of the three rock types after testing. The results from Ramsey and Chester^20^ and Bobich^21^ that the fracture angle increases with increasing confining stress provided the basis for the hypothesis that a hybrid failure mode exists when transitioning between extension and shear failure. For Longmaxi Shale, however, the fracture angle decreases with confining stress. The fracture angle variation is attributed to a combination of the dominant presence of bedding in Longmaxi Shale specimens and the variable stress distribution within the neck of the specimens. The understanding of a transition between tensile failure and frictional failure, however, remains valid. Figure 4 shows SEM images of typical micro-fractures in Longmaxi Shale after testing in the compressive region (higher confining stresses) and the tensile region (the lowest confining stress tested, σ_1_ = 0 MPa, σ_3_ = −5.5 MPa). These images evidence the lack of contact (larger fracture aperture) between fracture surfaces in the sample tested under the lowest confining stresses when compared to the sample tested in the compressive region.

When the confining stress is less than the σ_1_ threshold, cracks would not allow for frictional processes to dominate the strength of intact rock, and tensile strength will tend towards a constant value. This “tension cut-off” is observed for all three different materials, which indicates a universal characteristic for intact rock strength in the tensile region. Above this threshold, the linear trend of intact rock strength as function on confinement in the tensile region can be characterized by a coefficient of friction, μ, and the failure criteria becomes:

1 $${\rm{\sigma }}3\,{\rm{at}}\,{\rm{failure}}={{\rm{\sigma }}}_{t};\,{\rm{for}}\,{{\rm{\sigma }}}_{1} < ({\rm{\sigma }}t+{\rm{T}})/(1-{\rm{\mu }})$$

2 $${\rm{\sigma }}3\,{\rm{at}}\,{\rm{failure}}=(1-{\rm{\mu }})\,{{\rm{\sigma }}}_{1}-{\rm{T}};\,{\rm{otherwise}}$$

where T is the intersect of the linear trend of the data and the σ_3_ axis; and σ_t_ is the unconfined direct tensile strength (negative here). For the case of the three materials tested, the values of T and μ were 15.9 MPa and 0.23 for Longmaxi Shale, 24.7 MPa and 0.81 for Carrara Marble, and 15.7 MPa and 0.84 for Berea Sandstone. In Fig. 2, the vertical dashed line followed by the solid line for increasing σ1 represent the criteria presented here.

### Criteria applied to indirect tensile tests

**Figure 5:**
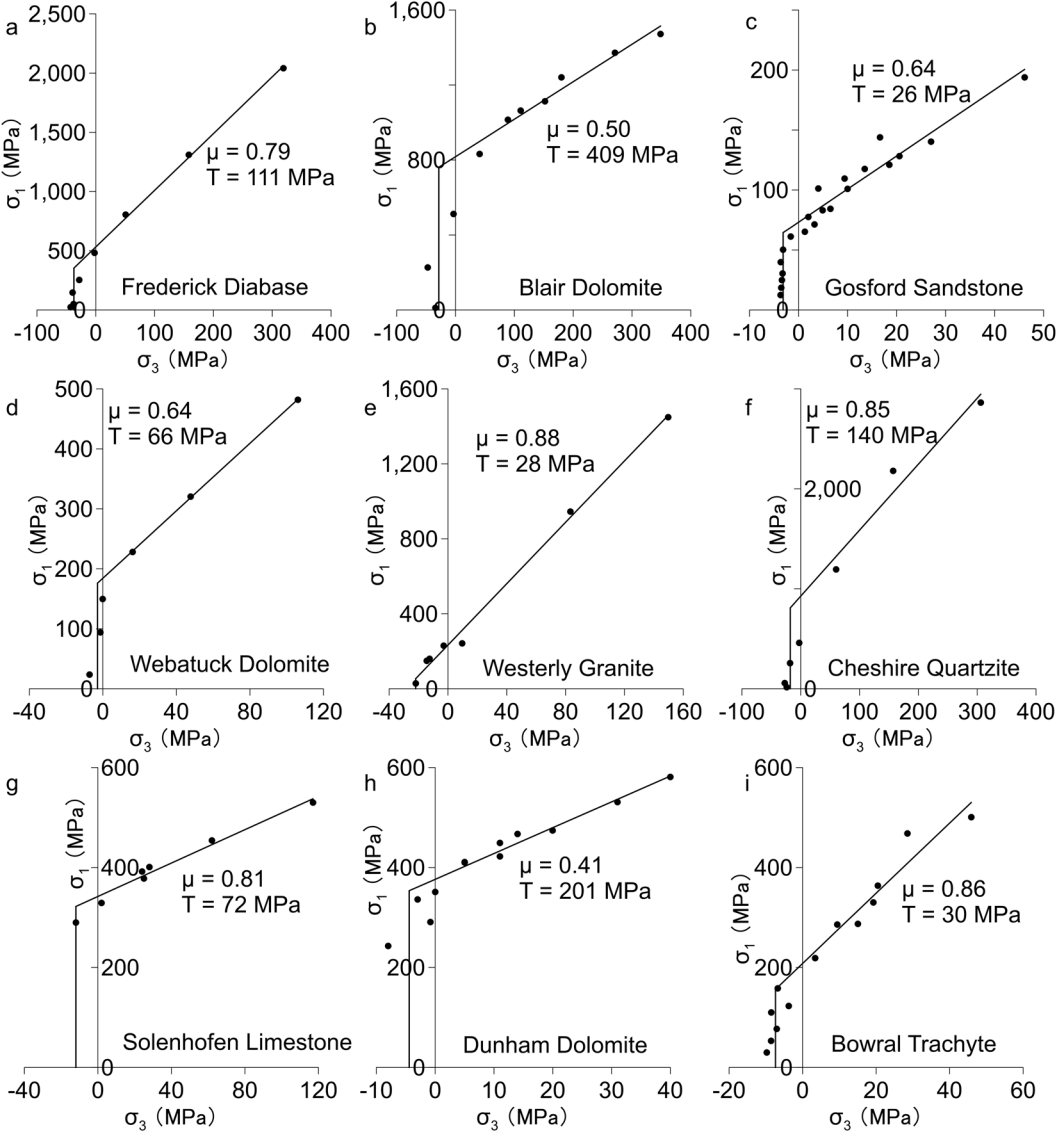
Tensile strength data^28–30^ and the fitted criteria presented in this paper. It can be observed that the criteria show general good agreement with the data (as per Fig. 2). Data for Blair Dolomite and Cheshire Quartzite appear to be overpredicted for the uniaxial compression, however data is scarce in these materials for a definite conclusion.

Tensile strength data was obtained from other direct tensile tests and from indirect tensile tests (Brazilian tests)^28–30^. The Brazilian test consists on compressing a disk of rock in the direction parallel to its flat surface, which induces tensile stresses at the center of the disk and perpendicular to the applied compression. The rock strength data is plotted in Fig. 5 and was used to assess the applicability of the criteria for other rock types and a wide range of strengths. Figure 5 also shows the strength criteria fitted for these rocks in the tensile region and the parameters T and μ for the fit. The data and the fit are in general agreement. This approach can be used as stand-alone within the tensile region and the transition towards the uniaxial compressive strength, or as a complement to the tension cut-off proposed in Hoek and Martin^13^.

## Discussion

The results of the analysis presented here show that: (1) friction processes have a significant effect on intact rock strength well into the tensile region. The strengths on Longmaxi Shale, Berea Sandstone and Carrara Marble then drop towards the unconfined tensile strength, which suggests that frictional processes do not dominate the strength of intact rock below a threshold value of confinement. This was evidenced by the Nano-scale SEM images, where unconfined tests presented larger crack apertures than confined tests, therefore precluding mobilization of frictional resistance. Moreover, the observation for Longmaxi Shale that most micro-cracks are distributed parallel to bedding^31^ also suggests bedding plays a significant role in the fracture process. Fracturing develops through bedding and through coalescence of micro-cracks within the tensile regime. This is illustrated by the rough fracture surfaces observed at low confining stresses in Longmaxi Shale. As confinement increases, shear strength along the bedding increases and the failure surfaces become smoother (Figure 3). (2) This behavior and the observations through SEM imaging prove the “tension cut-off” observed for brittle rocks occurs also in more ductile rocks like shales. This is understood as a transition to a state of low confinement stress, where fractures have larger openings and mobilized friction is reduced substantially such that it has a minor to no effect on the confined tensile strength of rock. The fact that tensile strength of shale behaves in a similar fashion as for brittle rocks, proves that tensile failure processes for intact rock are universal. Moreover, the effect of friction above a confinement threshold implies that even a minor increase in confinement stresses could mobilize frictional processes such that intact strengths in the tensile region could increase substantially, as is the case for Longmaxi Shale. This should be of particular interest to designers faced with the decision between leaving an intact rock structure unconfined and allocating resources to reduce the loss of confinement. This common behavior to intact rocks can be utilized for developing strength criteria that considers the frictional processes in the tensile region.

The tensile strength criteria presented here was derived with three comprehensive sets of confined, direct tensile tests on rocks that are known to show somewhat different behavior: shale, sandstone and marble. The criteria were tested for a set of 9 indirect tensile tests published in the literature, showing a wide range of strengths. The strength data and the fit for the criteria are in general agreement, however for two of the rock types where the criteria could overpredict the unconfined compression strength (Blair Dolomite and Cheshire Quartzite). However, data for these two rock types is scarce at the unconfined compressive strength for the indirect tensile test dataset and judgment on the adequacy of the criteria fit for these two rocks remains inconclusive. In general, the criteria are successful in representing the strength envelope in the tensile regime by representing the existence of a tension cut and considering a measure of the frictional processes (μ) and the amount of confinement (T).

The micro-fracture characteristics observed in the SEM images are consistent with our interpretation of the frictional processes leading to failure in the tensile regime and how they transition towards a purely tensional mode. This represents a first for failure of intact rock in the tensile region and sets an approach for further research into this phenomenon.

## Methods

We did tests on shales from Longmaxi Formation. It has extremely low porosity and small grains approximately less than 4μm in diameter. The grains include 31.8% to 42.7% quartz and 31.8% to 41.9% clay minerals (mainly illite), feldspar, dolomite, and pyrite according to the result of X-ray diffraction tests. The shales used in this study have very obvious bedding planes.

We follow the same test procedure and sample shape as Ramsey and Chester^20^ and Bobich^21^. The sample geometry referred to as the dog-bone geometry with a notch cut, consists of a cylindrical specimen with a reduced central neck. The purpose for this geometry was to produce the most reproducible and uniform stress conditions and predestine the location of failure within the center of the neck (i.e. at its smallest diameter)^22,23^.

The preparation of a dog-bone sample of shale is time-consuming due to its fragility under disturbance. This process starts with the rough grinding of 50 mm diameter shale cylinder approximately 120 mm long from a solid block of Longmaxi shale with a dimension of 300 mm × 300 mm × 300 mm. The laminae (bedding layers) of the samples are parallel to the axial of the cylinder. Next, two steps were taken during the sample fine grinding process to ensure that each sample meets quality standard. The neck portion of the sample is ground to 30 mm with the wheel radius of 90 mm. Then both ends of the sample are cut and ground perpendicular to the axis of the sample with a head depth of 9 mm. The target dimensions for all samples are 50 mm diameter for the cylinder ends, 30 mm diameter for the neck portion, and 90 mm radius of curvature. And the samples are 100 ± 0.85 mm in length, with a large head diameter of 50 ± 0.2 mm and neck diameter of 30 ± 0.67 mm.

All experiments for this study were conducted by the MTS 815. The MTS 815 is a closed-loop computerized triaxial testing apparatus with high-performance servo-hydraulic actuators. This MTS 815 apparatus is very suitable for extension experiments. First, the apparatus integral rigidity is 11.0 × 10^9^ N*m^−1^, and when used in combination with precisely machined samples, the generation of bending force in the samples is inhibited. Second, the MTS 815 is equipped with a high-resolution, digital data acquisition system. The maximum load capacity is 2600 kN with the measuring accuracy of ±0.5%.

The MTS 815 apparatus was redesigned to meet the requirement of the triaxial extension experiments: (1) build a synchronous chain between actuator and loading platform. Two steel plates linked to the loading platform are bolted to the actuator so as to ensure the displacement gauge in the actuator can detect the displacement change during the test. (2) sample fixture with multi-degree of freedom was developed. The fixture consists of the upper cushion block, lower cushion block, cardan and the pistons connecting to the cushion blocks. The cardan could provide a multi-variant adjustment to avoid the bending force in case of the misalignment between tensile direction and sample axial direction. New pistons were designed to glue samples and fasten to the end cup. Advanced Research Center in China South University is responsible for the modification.

The experiments were conducted at room temperature (28 °C) and humidity and extended axially at a rate of 2 × 10^−2^ mm*s^−1^. The whole experimental procedure involves 5 stages. First, 3 M™ Scotch-Weld™ Epoxy Adhesive 2216 is used to join sample and piston together, which requires more than 24 hours to maintain a strong bond. Then, a single polyolefin outer jacket is affixed to the piston and sample to prevent intrusion of the confining fluid. Next, a 0.1 kN tensile force is applied axially once the sample and piston are inside the vessel room, by pulling, in order for the specimen to be set in an adequate position before the testing start and no abrupt rearrangement of the specimen occurs. After that, the thick-walled pressure vessel is placed and the confining fluid is injected into the pressure vessel. The confining pressure is then set to the predetermined value. Application of tensile stress in the axial direction is achieved by displacing the loading platform such that the sample on the platform will be pulled until it breaks. A force gauge and a displacement gauge record the axial loads and displacement through the entire procedure.

## Data Availability

The data that support the findings of this study are available from the authors on reasonable request.
